# DDT exposure levels and semen quality of young men from a malaria area in South Africa

**DOI:** 10.1186/1475-2875-11-S1-P21

**Published:** 2012-10-15

**Authors:** Christiaan de Jager, Natalie H Aneck-Hahn, Maria S Bornman, Paulina Farias, Marcello Spanò

**Affiliations:** 1 UP Centre for Sustainable Malaria Control (UP CSMC), School of Health Systems & Public Health, University of Pretoria, Pretoria, 0001, South Africa; 2 UP CSMC, Department of Urology, University of Pretoria, Pretoira, South Africa; 3 Instituto Nacional de Salud Publica, Cuernavaca, Mexico; 4 Toxicology & Biomedical Sciences, ENEA Casaccia Research Center, Rome, Italy

## Background

The organochlorine pesticide DDT (1,1,1-trichloro-2,2-bis(chlorodiphenyl)ethane) has been used for malaria vector control in Limpopo Province, South Africa, since 1945. It is one of the Persistent Organic Pollutants (POPs) that are noted for their toxicity, persistence and bio-accumulative characteristics. Evidence of health effects in laboratory animals and wildlife are found in the scientific literature and include altered hormone activity [1]. DDT is still being used in many parts of the developing world for malaria vector control. Indoor spraying programmes in the Limpopo province include houses that are not painted on the inside. Exposure to DDT can be direct (spraying of houses) or indirect (through the food chain). DDT has got estrogenic properties and its degradation product, *p*,*p*’-DDE, is an anti-androgen [2]. In response to mounting concerns about the endocrine disrupting influence of environmental chemicals on human health, this epidemiological study was initiated in a malaria area where DDT is still used. The aim of the study was to investigate the DDT/DDE exposure levels, effects on seminal parameters and possible adverse effects on human sperm genetic integrity in a non-occupationally exposed population of young health men, living in a malaria area.

## Materials and methods

This cross-sectional study involved 209 young males recruited in an endemic malaria area (Limpopo Province, South Africa) where DDT is sprayed annually. DDT and DDE levels were measured in plasma. Semen analyses were done according to the WHO (1999) criteria. The flow cytometric sperm chromatin structure assay (SCSA) and Anilin Blue (AB) methods were used to assess sperm DNA/ chromatin integrity [3].

## Results

The lipid adjusted p,p’-DDT (mean±SD) concentration was 109.2±106.6 µg/g lipid whereas the p,p’-DDE concentration was 246.2±218.5 µg/g lipid. Several sperm motion parameters including the percentage of motile sperm were impaired with higher DDT and DDE concentrations (r=-0.27; p=0.001 and r=-0.20; p=0.001 respectively). Sperm motility and morphology were also negatively correlated with sperm DNA damage (-0.19; p=0.008 and -0.22; p=0.002). The results point to a weak association between DDT/DDE plasma concentration and the incidence of sperm with chromatin defects.

## Conclusions

The results suggest that non-occupational environmental DDT/DDE exposure have negative effects on seminal parameters and might impact on the sperm chromatin integrity and DNA damage of young South Africans. In response, the *University of Pretoria Centre for Sustainable Malaria Control* was established to make a sustainable contribution towards the creation of a malaria-free Africa. South Africa is working towards malaria elimination and is to facing many challenges regarding effective, safe and sustainable vector control methods.
